# Early Events in Chikungunya Virus Infection—From Virus Cell Binding to Membrane Fusion

**DOI:** 10.3390/v7072792

**Published:** 2015-07-07

**Authors:** Mareike K. S. van Duijl-Richter, Tabitha E. Hoornweg, Izabela A. Rodenhuis-Zybert, Jolanda M. Smit

**Affiliations:** Department of Medical Microbiology, University of Groningen and University Medical Center Groningen, 9700 RB Groningen, The Netherlands; E-Mails: m.k.s.richter@umcg.nl (M.K.S.D.-R.); t.e.hoornweg@umcg.nl (T.E.H.); i.a.rodenhuis-zybert@umcg.nl (I.A.R.-Z.)

**Keywords:** Chikungunya virus, alphavirus, cell tropism, receptor, entry, endocytosis, clathrin, fusion, neutralizing antibodies, entry inhibitors

## Abstract

Chikungunya virus (CHIKV) is a rapidly emerging mosquito-borne alphavirus causing millions of infections in the tropical and subtropical regions of the world. CHIKV infection often leads to an acute self-limited febrile illness with debilitating myalgia and arthralgia. A potential long-term complication of CHIKV infection is severe joint pain, which can last for months to years. There are no vaccines or specific therapeutics available to prevent or treat infection. This review describes the critical steps in CHIKV cell entry. We summarize the latest studies on the virus-cell tropism, virus-receptor binding, internalization, membrane fusion and review the molecules and compounds that have been described to interfere with virus cell entry. The aim of the review is to give the reader a state-of-the-art overview on CHIKV cell entry and to provide an outlook on potential new avenues in CHIKV research.

## 1. Introduction

Chikungunya virus (CHIKV) is an arbovirus transmitted by mosquitoes of the *Aedes (Ae.)* species. Upon infection, about 75%–95% of the individuals develop Chikungunya fever, characterized by high fever, myalgia, joint pain, rash, and intense asthenia [1,2]. A common long-term complication (occurring in 12%–49% of patients) is severe, debilitating joint paint that can persist for months to years after infection [3]. Furthermore, in rare cases, encephalopathy, encephalitis, myocarditis, hepatitis, and circulatory failure is seen [4,5].

Previously, CHIKV caused small outbreaks in confined regions within Africa and Asia. This situation drastically changed by the end of 2004 when the first major CHIKV outbreak started [6]. Since then, the virus has spread globally with millions of people infected. To date, CHIKV is epidemic in large parts of Africa, Asia, and the tropical regions of the Americas [7]. Within the last 1.5 years, the virus has spread to more than 40 countries within Central America involving over 1 million CHIKV infections [8]. There are four CHIKV lineages—the West African (WA) lineage, the Asian lineage, the Eastern/Central/Southern Africa (ECSA) lineage, and the Indian Ocean lineage (IOL); the latter emerged from the ECSA lineage in 2004 [9,10]. Some IOL strains adapted to a new vector, *Ae. albopictus*, without significantly compromising viral fitness for the initial vector *Ae. aegypti*, thereby increasing the epidemic potential of the virus. Functional studies revealed that this is caused by adaptive mutations within the viral spike proteins E1 and E2 of CHIKV [11,12]. The IOL lineage caused the majority of CHIKV outbreaks in 2004–2012, whereas the Asian lineage and ECSA lineage are mainly responsible for the current outbreaks in the Americas [10,13].

There is currently no vaccine nor a specific antiviral treatment available to prevent or treat CHIKV infection. A potential antiviral strategy involves the inhibition of the cell entry process of the virus. CHIKV cell entry is based on a series of dynamic events between the viral glycoproteins E1 and E2 and the host cell, including virus-cell attachment, virus internalization, intracellular trafficking, and membrane fusion. In this review we will describe the current knowledge related to the cell tropism, the cell entry pathway of CHIKV and will discuss the molecules that have been identified to interfere with these processes.

## 2. Viral Structure

CHIKV belongs to the alphavirus genus within the Togaviridae family. It is a member of the antigenic Semliki Forest Complex, which include, amongst others, the closely related O’nyong-nyong virus (ONNV), Semliki Forest virus (SFV), and Ross River virus (RRV). Other alphaviruses are for example Sindbis virus (SINV) and Venezuelan or Eastern Equine Encephalitis Virus (VEEV and EEEV, respectively) [14,15]. To date, most studies have been performed with SFV, SINV, RRV, and VEEV. Alphaviruses are enveloped spherical particles with a diameter of 65–70 nm [16,17]. The alphavirus genome consists of a single-stranded positive-sensed 11.8 kB RNA molecule packaged by the C protein to form the nucleocapsid. This nucleocapsid is surrounded by a host-cell derived lipid bilayer with two inserted transmembrane glycoproteins, E1 and E2 [15]. The composition of the host-cell derived lipid bilayer strongly resembles the plasma membrane of the infected host cell. For mammalian-derived CHIKV virions, the membrane consists of cholesterol and phospholipids in a ratio of approximately 1:1 [18,19,20,21].

**Figure 1:**
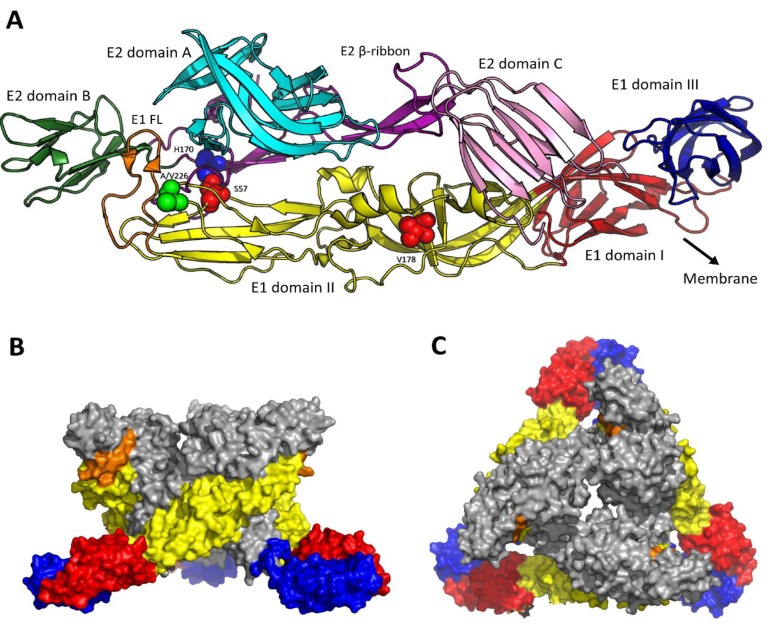
Structure of the E2/E1 dimer. (**A**) Ribbon diagram showing the ectodomains of the CHIKV E1 and E2 glycoprotein ([22]; PDB 3N41). The structural domains I, II, and III of E1 are shown in blue, red and yellow, respectively. E2 domain A, B, and C are designated in cyan, green, and pink, respectively. In the mature virion, the E1 fusion loop (E1-FL, orange), is covered by a binding groove between E2 domain A and B. The β-ribbon of E2 containing the acid-sensitive region is highlighted in dark purple. Within this region, the hydrogen bond between E2-H170 and E1-S57 stabilizes the E2/E1 dimer interaction at neutral pH [26,142]. E1-A/V226 and E1-V178 are important for lipid sensing before fusion [12,154]. The black arrow points towards the viral membrane; (**B**,**C**) Surface view (PDB 2XFC) of one virus spike from the side (**B**) and the top (**C**). E1 is depicted in the same colors as in the ribbon diagram, E2 is depicted in gray for clarity. This figure was prepared using the program PyMOL.

The E1 protein is 439 amino acids (aa) long and contains one conserved N-linked glycosylation site at position 141 [22]. E1 is anchored in the lipid bilayer with a 30 residue transmembrane helix at the carboxy-terminal end. The cytosolic region is only five residues in length and does not interact with the nucleocapsid [22,23]. The N-terminal ectodomain of E1 consists of 404 residues and is structurally divided into three β-barrel domains named DI, DII, and DIII (Figure 1a).

DIII is situated at the C-terminus of the protein and closest to the envelope, followed by the central DI and DII at the tip, which contains a hydrophobic fusion peptide [22,24,25,26]. The E2 protein has a length of 423 aa and is N-glycosylated at positions 263 and 345 [22]. Blast analyses revealed that like for E1, the glycosylation sites of E2 are conserved between all four CHIKV lineages (GenBank accession numbers strain RSU1: HM045797.1; strain IbH35: HM045786.1; strain S27: AF369024.2; strain LR2006 OPY: DQ443544.2). At the C-terminus, a transmembrane helix of 26 residues is located, followed by a cytoplasmic domain of 33 residues. The cytoplasmic domain contacts the nucleocapsid and studies with other alphaviruses have shown that this interaction is important for the correct assembly and budding of progeny viruses from the plasma membrane of infected cells [22,27,28]. The ectodomain of E2 has a size of 364 aa and consists of three immunoglobulin-fold domains termed A, B, and C, which are connected by a long β-ribbon (Figure 1a) [26,29].

On a mature virion, 240 copies of E1 and E2 are arranged as 80 trimeric spikes; a single spike consisting of three E2/E1 heterodimers (Figure 1b,c). The spikes are positioned in an icosahedral T = 4 symmetry and form a continuous protein shell around the particle [22,26,30,31,32]. Within the E2/E1 heterodimer, E1 laterally contacts E2 along the central domain II and partially domain III. The E1 hydrophobic fusion peptide is buried in a groove between domain A and domain B of E2 (see Figure 1), thereby preventing pre-mature activation of the membrane fusion machinery of the virus [26].

## 3. Viral Tropism

Infection starts when a CHIKV-infected *Ae.* mosquito is feeding on a human host [33]. During feeding, CHIKV particles are thought to be released within the dermis and into the subcutaneous capillaries of the skin [34]. Within 2–4 days, the virus reaches the blood and disseminates to other parts of the body. Although CHIKV pathogenesis is still poorly understood, recent studies shed light onto the organs and cells involved in CHIKV replication (systematically reviewed by [35]). The CHIKV target organs include joints, muscle, skin, and less frequently, the liver, kidneys, eye and the central nervous system (CNS). Infection of these organs is frequently associated with a marked infiltration of mononuclear cells such as monocytes/macrophages. The virus tropism described within this section is mostly based on studies using ECSA and IOL strains. A few studies directly compared the infectivity of IOL, WA and ESCA on multiple cell lines and revealed that these viruses exhibit a comparable tropism [36,37,38]. However, more studies are required to determine the exact tropism for all four CHIKV lineages.

### 3.1. Viremia—Where Is the Virus Produced?

During the 7–12 days-long acute viremic period, CHIKV load can reach 10^9^–10^12^ viral particles per milliliter [39,40,41]. The observation that CHIKV reaches a high titer in a relatively short time period is suggestive for replication in blood leukocytes [42]. Indeed, other alphaviruses replicate in immune cells including dendritic cells (e.g., SFV, RRV, and VEEV) and monocytes (e.g., RRV and VEEV) [36,43,44,45,46]. In contrast to the above-mentioned alphaviruses, peripheral blood mononuclear cells (PBMCs) do not seem to contribute significantly to the production of CHIKV progeny [36,47]. In fact, *in vitro* analysis revealed that most blood-derived cell types such as lymphocytes, dendritic cells, and natural killer cells are refractory to CHIKV infection [36,37]. Conflicting reports were published on the permissiveness of monocytes to CHIKV infection [36,42]. However, it is clear that even though monocytes might harbor CHIKV antigens, viral production supported by the primary cultures of monocytes cannot explain the titers detected in blood of acute phase patients. These observations suggest that local CHIKV replication in dermal fibroblasts, migrating monocytes/macrophages, and endothelial cells are pivotal for virus production. Indeed, *in vitro* studies revealed these cells are much more permissive to CHIKV infection [36,37,48,49].

### 3.2. Arthrotropism of CHIKV

Mononuclear cell infiltration and viral replication in the muscles (particularly skeletal muscle progenitor cells, not muscle fibers) and joints (in fibroblasts of the joint capsule and presumably in osteoblasts) are associated with debilitating arthralgia, myalgia, and in some cases, arthritis [50,51,52,53,54,55]. While the acute phase symptoms usually resolve within two weeks, the musculoskeletal pain may linger for weeks to months or even years [56,57,58,59,60]. Chronic disease has been linked to persistent virus replication in the target cells and/or the establishment of a self-sustained inflammatory mechanism that leads to the tissue damage (for more details on this topic see [3,61,62]). *In* *vivo*, synovial macrophages and satellite muscle cells have been shown to contain viral RNA or protein months after infection [63,64]. However, the exact mechanisms underlying CHIKV persistence in tissue sanctuaries are still ill-understood and more studies in animal models and clinical investigations are needed to address this issue.

### 3.3. Less Common Tropism of CHIKV

CHIKV is classified as an Old World arthritogenic alphavirus and hence is not expected to be neurotropic or encephalitogenic [14]. However, cases of Guillain-Barré syndrome and encephalitis have been reported following CHIKV infection [65,66,67]. CHIKV and anti-CHIKV IgM has been detected in the blood-cerebrospinal fluid of human neonates and adult patients with encephalopathy [68]. Studies in mice suggest that CHIKV particles can enter the CNS via the Virchow-Robin spaces and choroid plexuses [55]. Thereafter, replication occurs within choroid plexus epithelial cells, leptomeninges, and ependymal cells but not in brain parenchyma [4]. *In vitro*, however, parenchymal cells including neurons, astrocytes, microglial cells, and neuroblastoma cells were found to be permissive to all strains tested (including ECSA, WA, and IOL) [4,37,38,69,70,71]. The ability of CHIKV to infect brain endothelial cells is still under debate as human brain microvascular endothelial cells can be infected, while primary brain endothelial cells [55] and the brain endothelial cell line hCMEC/D3 [36] were found to be refractory to infection by IOL isolates. The mechanism leading to CHIKV infection of the CNS in humans is to be elucidated.

## 4. Cell Entry and Membrane Fusion

### 4.1. Receptor Binding

The first step in infection involves binding of the virus to a host cell receptor [72,73]. Based on the wide range of cell types CHIKV infects *in vivo* and *in vitro*, the cellular receptor of CHIKV is likely to be ubiquitously expressed among species and cell types. Receptor binding is facilitated by the E2 glycoprotein of CHIKV [74,75]. Both domain A and domain B of the E2 protein contain putative receptor binding sites [26,29]. Furthermore, bioinformatic analysis revealed that E2 domain B contains a class III PDZ binding motif [76]. These motifs have been described to mediate protein-protein interactions [77,78].

To date, prohibitin (PHB), phosphatidylserine (PtdSer)-mediated virus entry-enhancing receptors (PVEERs), and glycosaminoglycans (GAGs) have been suggested as CHIKV receptor proteins in mammalian cells [69,79,80] and ATPsynthase β subunit in mosquito cells [81]. Notably, CHIKV infection can proceed in absence of these proteins, indicating that these proteins facilitate the initial interaction with the cell surface rather than virus uptake [82,83].

#### 4.1.1. Prohibitin

Prohibitins (PHBs) are evolutionary conserved multifunctional membrane proteins, which are present in multiple cellular compartments [84]. PHBs play a role in for example mitochondrial integrity, cell proliferation, cell survival and endocytosis in white adipose tissue [85,86,87]. Importantly, PHBs are ubiquitously expressed at the cell surface of numerous mammalian cells [77,78]. Wintachai and co-workers showed that anti-PHB antibodies and siRNAs towards PHB reduced CHIKV infection of microglial cells up to two-fold. CHIKV was also found to bind to PHB in U937 cells, but despite this interaction the cells did not support a productive infection [69]. On the other hand, flavaglines, plant compounds that directly interact with PHB did inhibit CHIKV infection in HEK-293T cells for up to 50% [88]. Thus, it is clear that PHB facilitates virus-cell binding. PHB likely acts to capture and concentrate CHIKV particles at the cell surface. However, since the inhibiting compounds only moderately reduced infectivity and that U937 cells are refractory to CHIKV despite PHB binding demonstrates that other factors are required to mediate (efficient) infection. The precise role of PHB in CHIKV cell entry remains to be elucidated.

#### 4.1.2. Phosphatidylserine (PtdSer)-Mediated Virus Entry-enhancing Receptors

T-cell immunoglobulin and mucin domain (TIM) family members are expressed on various immune cells and a range of mucosal epithelia, and are known to regulate immune cell activity [89,90,91,92]. Recently, TIM-1 was described to enhance the entry and infection of chimeric virus particles displaying the glycoproteins of CHIKV or other viruses in HEK293T cells [79]. TIM-1 binds to phosphatidylserine (PtdSer) in the viral envelope and functions to concentrate the virus at the cell surface. These receptors act on the basis of their long stalk region and PtdSer binding motif. Indeed, other unrelated proteins with a long stalk region and PtdSer motif were also able to support viral cell entry, demonstrating that virus-cell binding is not TIM-1 specific [93]. These results further indicate that CHIKV uses TIM-1 as an attachment factor but not as a specific receptor.

#### 4.1.3. Glycosaminoglycans

Glycosaminoglycans (GAGs) are large complex carbohydrate molecules that are expressed at the cell surface of most mammalian cell types. GAGs include among others heparan sulfate, keratan sulfate, chondroitin sulfate, and dermatan sulfate [86]. These molecules can bind a wide variety of proteins and mainly function in cellular adhesion, growth, differentiation, and signaling [94]. Several alphaviruses are known to use GAGs for cell entry [95,96,97,98,99]. Natural isolates of EEEV and low passage strains of VEEV were found to depend on GAGs for efficient infection of cells [97,100]. For other alphaviruses, heparan sulfate binding was related to virus-cell culture adaptation [95,96,101] and an attenuated disease phenotype in mice [95,96].

For CHIKV, GAG expression was found to increase the binding and infection efficiency of both a clinical and a vaccine strain in CHO cells. However, GAG binding is not a property of all CHIKV strains, as CHIKV-LR replicon particles do not require cell-surface GAGs for infection [80,102]. Yet, CHIKV like other alphaviruses readily adapts to GAGs [103,104]. GAG utilization is facilitated by mutations to positively charged amino acids at E2-82 and E2-79 [80,104]. For example, an arginine at E2-82 or a lysine at E2-79 leads to enhanced infectivity in mammalian cells and attenuated virulence in mice [75,104,105]. Though, the observation that a clinical strain utilizes GAGs for cell entry suggests that GAGs might also play a role in natural infection.

#### 4.1.4. ATP Synthase β Subunit

ATP synthase β subunit (ATPSβ) was recently found to interact with CHIKV in mosquito cells. Furthermore, ATPSβ-down-regulation significantly reduced viral entry and virus production [81]. The ATPSβ gene is widely conserved and is for example expressed in human endothelial and hepatic cells [106,107,108]. Although involved in F1/ATPase catalysis in the mitochondria, ATPS is also located at the surface of the plasma membrane. There, it can bind ligands as apolipoprotein A-I, apolipoprotein E and angiostatin [94]. Therefore, it is of interest to examine whether this protein is involved in CHIKV entry in mammalian cells and whether ATPSβ also exerts its function via increasing attachment of virions to the cell surface or whether other mechanisms are involved.

#### 4.1.5. Other CHIKV Receptors Candidates

Another potential CHIKV receptor is the aV integrin (ITGAV) and b1 integrin (ITGB1) dimer, consisting of two members of the integrin superfamily. Integrin superfamily members form various transmembrane dimers, which function as cell adhesion receptors binding to different extracellular ligands [109]. The aV integrin (ITGAV) and b1 integrin (ITGB1) dimer was found to be differentially expressed in the brain proteasome of mice early in CHIKV infection. A direct effect of the integrin dimer on CHIKV infection has not been studied yet [110]. However, these protein dimers were previously reported as an adenovirus receptor [111,112] and other members of the integrin superfamily serve as receptors for RRV and West Nile virus [113,114]. As rare cases of neuropathology have been described, it would be of interest to investigate if CHIKV infection of the CNS is facilitated by integrin dimers.

Additionally, Heat shock protein 60 (HsP60) is a CHIKV receptor candidate. HsP60 is mainly known as a mitochondrial molecular chaperone which is involved in protein folding [115]. The protein has also been detected at the cell surface of murine monocytes/macrophage, B lymphocytes and T lymphocytes and human T lymphocytes [116,117]. HsP60 was found to interact with CHIKV by a two dimensional Virus Overlay Protein Binding Assay (2D-VOPBA) [69,118]. HsP60 was previously implicated in DENV infection [119], but, thus far, no functional proof on the role of HsP60 in CHIKV entry has been explored.

### 4.2. CHIKV Cell Entry and Membrane Fusion

**Figure 2:**
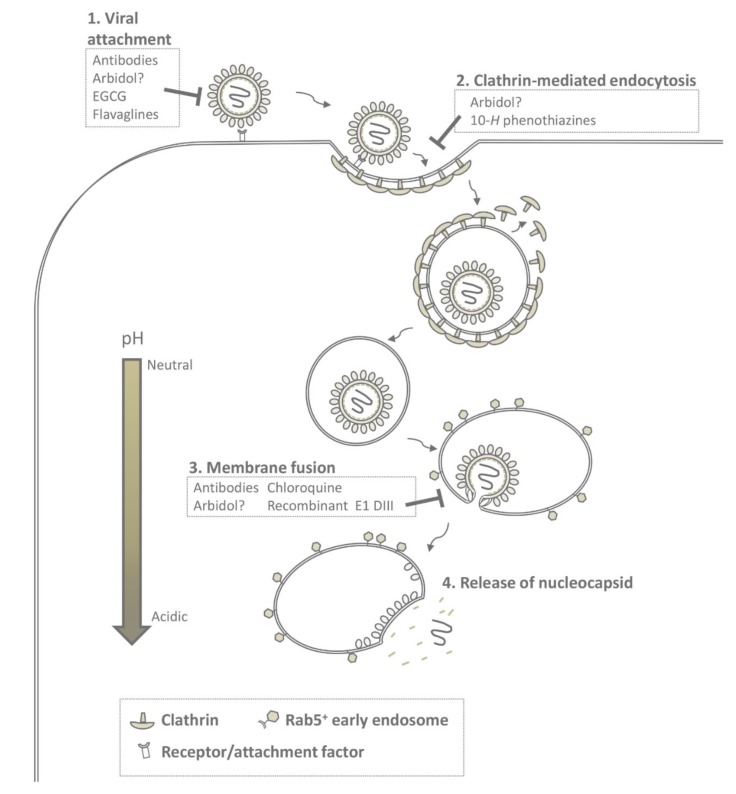
Chikungunya virus cell entry and potential antiviral strategies. The viral life cycle starts with attachment of the virus particle to one of the ubiquitously expressed attachment factors or receptors at the cell surface (**1**); Subsequently, the virus is internalized into the cell via clathrin-mediated endocytosis (**2**); Then, clathrin-molecules dissociate from the vesicle and the virus is delivered to Rab5^+^ endosomes. Within the mildly acidic lumen of the endosome, the viral glycoproteins E2 and E1 undergo major conformational changes that lead to membrane fusion (**3**); Thereafter, the nucleocapsid core is released into the cytosol (**4**). The molecules and compounds that are known to interfere with entry are stated in the boxes.

Alphaviruses are generally internalized via clathrin-mediated endocytosis (CME), though also direct fusion with the plasma membrane has been described for SINV [120,121]. CME is a constitutive process within mammalian cells [82,122]. Invagination and scission of the membrane to form a virus-containing clathrin-coated vesicle occurs via a complex interplay of several proteins including adaptor protein-2, dynamin, clathrin, epsin, and Eps15 (see for extensive review [123]). Thereafter, the clathrin-coated vesicle is transported inside the cell after which the clathrin molecules dissociate and the virus is delivered to endosomes. The low-pH environment of the endosomes subsequently triggers conformational changes within the E1/E2 glycoproteins to mediate fusion of the viral membrane with the endosomal membrane (see Figure 2). Alphavirus fusion usually occurs from within mildly acidic early endosomes. VEEV fusion on the other hand has been described to fuse from within late endosomes [124,125,126,127].

For CHIKV, contradicting observations were reported. CHIKV infection was found to be dependent on dynamin [36], a large multidomain GTPase driving the pinching of endocytic vesicles from the plasma membrane [123]. Dynamin is an important mediator of CME and caveolar endocytosis [127]; and is also described to act in phagocytosis [128]. Furthermore, CHIKV infection was found to be mediated by Eps15 [129], a molecule essential for the assembly of the clathrin-coated pits [123]. Involvement of Eps15 can however not confirm entry via CME, as Eps15 has also been implicated in clathrin-independent entry pathways [130]. Specific inhibitors like siRNAs against the clathrin heavy chain did not inhibit CHIKV infection in HEK239T cells [129], but did show a marked reduction in infectivity in human umbilical vein endothelial cells (HUVEC), the cell line U-2 OS, and primary human umbilical vein endothelial cells [131]. Moreover, we recently showed that Pitstop2, a biochemical inhibitor of CME, reduced CHIKV cell entry in BS-C-1 cells [132]. Furthermore, using live-cell microscopy, we determined that approximately 90% of all particles that fused entered through CME. Taken together, thus far, a limited number of studies has been performed to investigate the cell entry pathway of CHIKV and most of these studies point towards entry through CME although clathrin-independent entry is also reported. The entry pathway taken by the virus maybe cell-specific. Alternatively, CHIKV has the capacity to infect cells via multiple pathways. The latter is supported by the fact that none of the inhibitor strategies applied so far completely blocked CHIKV infection [36,129,131,132]. In the absence of cellular perturbations, CHIKV was shown to enter via CME and therefore we hypothesize that CME, like for other alphaviruses, is the main pathway exploited by CHIKV.

Upon endocytosis, the virus is delivered to early endosomes. Approximately 40% of the particles fuse within 10 seconds after delivery to the endosome. More than 95% of all CHIKV fusion events occurred from within in early endosomal compartments [132]. This is in line with data from Bernard *et al.* who showed that CHIKV infection is dependent on early endosomes, but not on late endosomes [129]. In mosquito cells, however, CHIKV infection was dependent on the integrity of both Rab5 and Rab7-positive endosomes, which is suggestive of fusion from within maturing or late endosomes [133]. Variability in endosomal pH between cells [134] may explain this discrepancy. Biophysical analysis revealed that the pH threshold for CHIKV fusion lies—depending on the viral strain—between pH 6.2 and 5.9 [132,135]. Thus, we postulate that fusion is triggered once the pH of the endosomal lumen is below the threshold for membrane fusion. It is highly unlikely that other processes are involved, especially considering the rapid kinetics of membrane fusion within Rab5-positive endosomes.

## 5. Molecular Mechanism of CHIKV Fusion

**Figure 3:**
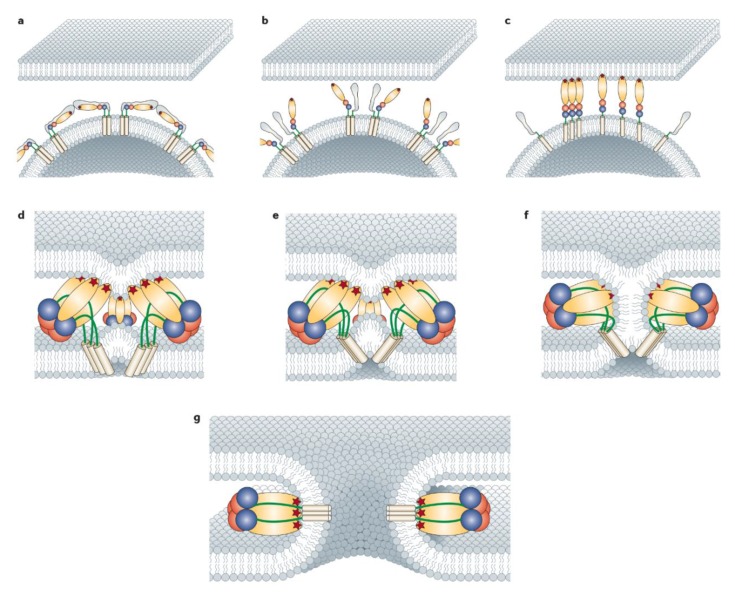
Model of alphavirus membrane fusion [145]. (**a**) On a mature virion, 240 copies of E1 and E2 are arranged as 80 trimeric spikes; a single spike consisting of three E2/E1 heterodimers. Domains of E1 are colored as in figure 1; E2 is shown in gray. The E1 hydrophobic fusion loop (indicated as a star) is buried in a groove between domain A and domain B of E2; (**b**) Destabilization of the E2/E1 heterodimer is triggered once the virus is exposed to the mildly acidic pH. Domain B of E2 moves away and the E1 fusion loop is exposed; (**c**) Insertion of the fusion loop into the target membrane and dissociation of the E2 protein. Formation of a E1 core trimer between DI and DII; (**d**,**e**) Re-folding of E1 DIII and stem region to form a hairpin-like homotrimer, forcing the two opposing membranes together; (**f**) Merging of the opposing membrane leaflets (hemifusion); (**g**) Formation of the final stable homotrimer and opening of the fusion pore. (Figure reprinted with permission from Nature Reviews Microbiology).

The molecular mechanisms involved in the membrane fusion process have been studied in great detail for SFV and SINV. The studies published thus far on CHIKV suggest that the molecular mechanisms involved in fusion are highly conserved between alphaviruses [26,135,136]. For example, like SFV and SINV [137,138,139], CHIKV can fuse with receptor-free liposomes, indicating that fusion is independent of a protein receptor [132,135]. The fusion process can be roughly divided in the following steps: (1) destabilization of the E2/E1 heterodimer, (2) integration of the E1 protein in the target membrane, (3) E1 trimerization, and (4) fusion pore formation (Figure 3).

Destabilization of the alphavirus E2/E1 heterodimer is triggered once the virus is exposed to the mildly acidic pH within the endosomes [26,140]. Histidines, which have a pkA of ~6–7, play a critical role in this process [25,141]. Recently, and in line with other alphaviruses [25], a series of highly conserved histidines within the envelope glycoproteins of CHIKV has been identified to control the pH-dependent conformational changes during fusion [136]. For example, E2-H170, a residue that is located within the acid sensitive region (ASR) of E2 (Figure 1, purple), becomes largely disordered at low pH [26,142]. Earlier work on SFV showed that protonation of this residue reduces the stability of the E2/E1 heterodimer by disabling the hydrogen bond with E1-S57. Once the E2/E1 interactions are loosened, the B domain of E2 moves away and the E1 fusion loop is exposed [26,29,143]. Thereafter, the E1 protein adopts an extended form and the hydrophobic fusion loop inserts into the target membrane (Figure 3) [17,144,145]. For SFV, this interaction is both low pH and cholesterol-dependent [144,146,147]. The presence of sphingomyelin strongly stimulates cholesterol- mediated E1 binding, but is not strictly required [147,148]. It is likely that these lipid interactions are similar for CHIKV, as cholesterol and sphingomyelin in the target membrane greatly enhance the fusion potential of CHIKV [132,135]. One of the amino acids important for lipid- and pH-sensing of SFV, SINV and CHIKV is situated at the E1-226 position [12,149,150,151]. This residue lies within the central DII domain in close proximity to the fusion loop (Figure 1) [25]. CHIKV strains with a valine instead of an alanine at the 226 position are more dependent on cholesterol and require a lower pH for infection [12,152] and fusion [132,153,154]. Another residue that has been reported to play a role in SFV lipid recognition is E1-V178 [155,156]. This residue is conserved among most alphaviruses, and experimental mutation of this residue to alanine leads to decreased cholesterol dependence of CHIKV fusion [154].

When the fusion peptide inserts into the target membrane, E2 is presumably still in association with the E1 molecules, as has been shown for SINV [17]. As the pH further decreases, the E2 molecules completely dissociate, which enables E1 trimerization [17,157]. A highly conserved histidine residue (E1-H3) is essential in regulating low-pH-induced trimerization [136,158,159]. For SFV, the first step in E1 trimerization involves the formation of a core trimer between DI and DII, which is dependent on low pH and most likely the presence of cholesterol and sphingomyelin in the target membrane [147,160,161,162]. Furthermore, sphingolipids have been proposed to play a role in stabilizing the E1 trimer [155]. After formation and stabilization of the core trimer, domain III re-folds back independently of pH towards the core trimer to form a hairpin-like homotrimer (Figure 3) [160,161]. This process brings the two opposing membranes together and forces merging of the outer membrane leaflets (hemifusion). Subsequently, a fusion pore is formed and expands, through which the nucleocapsid gains access to the cytosol [160,163]. For SFV, it has been shown that several E1 homotrimers assemble in a ring-like structure on the target membrane [158,160] with recent research indeed suggesting that for CHIKV fusion, several trimers need to act simultaneously to mediate membrane fusion [135].

## 6. Inhibition of Early Events in Infection

Many studies have focused on the development of antiviral drugs to CHIKV. Antiviral treatment to reduce viremia is probably only possible in areas with hyper-endemic CHIKV activity. This as treatment should start quickly after disease onset given the short period of viremia. The development of antivirals is important as it may prevent the development of persistent disease. Indeed, mouse studies have indicated that antibody-based therapy might prevent persistent infection. Thus far, multiple inhibitors have been identified affecting different stages of the viral life cycle (extensively reviewed in [1,164]). Here we will discuss the compounds that specifically interfere with: (1) attachment of the virus to the target cell, (2) endocytosis, and (3) membrane fusion.

### 6.1. Interference with Virus-Receptor Binding

The green tea component epigallocatechin-3-gallate (EGCG) was found to inhibit CHIKV attachment and infection of HEK293T cells [165]. EGCG has a broad antiviral activity against numerous viruses, and presumably acts via binding competition with heparan sulfates and sialic acid [166]. Other plant-derived compounds that interfere with virus-receptor binding are flavagline, which act by binding to the CHIKV attachment factor PHB [88].

Another strategy involves the use of neutralizing monoclonal antibodies (MAbs) [167,168]. For CHIKV, the MAbs CHK-9, m242, and IM-CKV063 have been described to target the E2 putative receptor-binding domain A and have been proposed to prevent cellular binding [22,169]. Blocking infection through interference of virus-receptor binding is however challenging as CHIKV interacts with multiple attachments factors via distinct epitopes on E2 domain A and B. Furthermore, antibody-binding to CHIKV particles will target the immune-complex to Fc receptor-expressing cells which internalize the particle via interaction of the antibody to the Fc receptor. It remains to be investigated if antibodies that interfere with virus-receptor binding neutralize CHIKV infection in cells expressing the Fc receptor.

### 6.2. Interference with Endocytosis

In a large screen using a CHIKV replicon system, compounds with a 10-*H* phenothiazine structure including the licensed antipsychotic drug chlorpromazine were found active against CHIKV [170]. Chlorpromazine has been implicated to block the formation of clathrin-coated pits [171]. Since it is likely that CHIKV infects cells via this pathway it is of interest to evaluate if compounds with a 10-*H* phenothiazine structure have potential in antiviral treatment. However, since chlorpromazine is prescribed for various psychotic disorders, the psychological effects of these compounds should be monitored closely. In addition, single molecule therapy seems unlikely as CHIKV was found to enter cells via clathrin-dependent and clathrin-independent pathways. The anti-malaria drug chloroquine, which inhibits acidification of endosomes, was also found to hamper CHIKV infection [172,173,174,175]. Unfortunately, however, a double-blind placebo-controlled randomized clinical trial showed that chloroquine-treatment does not reduce viremia or the frequency of febrile arthralgia. In fact, an increased prevalence of persistent arthralgia was seen compared to the control group [172]. Given these results it seems doubtful that chloroquine-based therapy will be pursued in future studies.

### 6.3. Interference with Membrane Fusion

A pivotal step in membrane fusion is re-folding of the E1 DI/DII core trimer against the DIII stem [160,161]. Indeed, binding of exogenous recombinant E1-DIII proteins of SFV and CHIKV efficiently inhibit CHIKV membrane fusion and infection of BHK cells. Here, the presence of the stem region in the exogenous CHIKV E1-DIII proteins was a prerequisite for inhibition of membrane fusion [162]. Therefore, like for other alphaviruses, the E1 stem region has been proposed as a candidate target for small molecule inhibitors against CHIKV [162,176]. However, no specific drug that acts on this level has been described so far.

Another group of compounds that have been described to possess potent antiviral activity to CHIKV are arbidol (ARB) and it derivatives [177,178]. Arbidol (ARB) was originally licensed in Russia to treat and prevent Influenza infections. Escape mutant analysis and attachment assays revealed that ARB interferes with the early stages of CHIKV infection [177]. The precise mechanism underlying ARB activity remains to be elucidated, but earlier studies independent of CHIKV postulated that ARB functions through inhibition of virus-membrane fusion [179,180].

An alternative strategy to block membrane fusion is the use of MAbs. CHIKV MAb IM-CKV063 has been suggested to stabilize the E2/E1 trimeric spike as it binds to a conformational epitope spanning two E2 units [169]. Furthermore, the strongly neutralizing MAb C9 was found to target the ASR of E2 and has been predicted to prevent the conformational changes preceding membrane fusion [181]. MAb CHK-152 was shown to stabilize the E2 B domain, thereby inhibiting the exposure of the E1 fusion loop [22]. Indeed, functional studies revealed that CHK-152 abolishes membrane fusion activity of CHIKV. Importantly, this antibody also showed protective efficacy in mice and therefore is a candidate for antiviral therapy [182]. Thus, a variety of antibodies have been identified to interfere with infection and future studies should address which cocktail of antibodies has the largest therapeutic potential.

## 7. Future Perspectives and Concluding Remarks

Since the re-emergence of CHIKV about a decade ago, our understanding of the biology of the virus has greatly improved. Important progress has been made in defining the cells targeted and the pathways exploited by the virus to enter these cells. However, more research is required to fully elucidate the mechanisms of CHIKV infection, as some steps remain unclear.

For example, it will be important to identify the cells that serve as the main viral factories during the viremic period. Identification of the cells that produce most virus progeny will not only increase our understanding of CHIKV pathogenesis but will also guide the development of antiviral treatments. Furthermore, although we know that persistent CHIKV replication is associated with chronic arthralgia, the understanding of the underlying mechanism is poor. However, a growing body of evidence points towards an imperative role of an altered immune response initiated during acute infection in the development of chronic disease [55,62,183,184,185]. More *in vivo* studies in animal models will be required to gain more insight into the correlation of CHIKV tropism and viral persistence.

To date, several molecules have been described to facilitate CHIKV infection. However, most if not all molecules act as an attachment factor rather than an entry receptor. The entry receptor pivotal for CHIKV infection has yet to be identified. On the other hand, one can question whether such an entry receptor exists, considering the broad range of cells that can be infected and the fact that no receptor is required for membrane fusion. Based on the studies conducted thus far it becomes clear that CHIKV can utilize a variety of attachment factors and this may be sufficient to enter a cell.

The membrane fusion machinery of alphaviruses is largely defined. However, further fine-tuning is warranted as it may guide the development of compounds that interfere with infection. Mutagenesis studies already have enormously increased our understanding of the membrane fusion mechanism and will continue to do so. For dengue, an antibody has been identified that “traps” a fusion intermediate [186], and using autologous CHIKV antibodies represents another useful approach to further unravel the viral fusion mechanism.

CHIKV enters cells via clathrin-mediated endocytosis and fuses from within early endosomes. Interestingly, novel adaptations in the emerging IOL strains like the A226V mutation in E1 and substitutions in the acid-sensitive region of the E2 protein alter the pH-dependent membrane fusion properties of the virus. Furthermore, increased infection of mosquito midgut cells was observed, which likely led to the enhanced fitness of the virus in *Ae. albopictus* [153]*.* It will be interesting to investigate if there is a direct correlation between the higher infection rate and the altered pH-dependent membrane fusion properties. For example, the site of membrane fusion may be important for successful initiation of infection.

The only entry inhibitor tested so far in a clinical study, chloroquine, gave disappointing results [172]. Ongoing discovery of antiviral inhibitors is of utmost importance given the high burden of CHIKV infection. Antibody-based therapy is an attractive approach especially as it has been shown that it might prevent persistent infection in mice [187]. The proof-of-principle that antibody-based therapy is effective in humans is expected soon as a current clinical trial is evaluating the effect of anti-CHIKV serum antibodies in preventing severe disease in neonates [188]. This group of patients is of special interest for treatment as newborns are more likely to develop severe disease and can be treated during early stages of disease [1]. Antibody-based therapies likely always consist of a set of antibodies. This because CHIKV contains multiple receptor binding domains and it is unlikely that one antibody abolishes virus-receptor interaction in all CHIKV permissive cells. Furthermore, antibody-bound particles are internalized via Fc-receptors expressed on immune cells like macrophages, cells that facilitate CHIKV infection [37]. We hence postulate that antibodies targeting the membrane fusion machinery of the virus represent the most robust entry inhibitors. Therefore, focus should be laid on the further identification of neutralizing antibodies that interfere with membrane fusion.
